# Community-level determinants of buprenorphine provider availability: A ZCTA-level (zip code tabulation area) analysis

**DOI:** 10.1016/j.dadr.2026.100476

**Published:** 2026-09-04

**Authors:** Sadia Jehan, Kristen D. Seay

**Affiliations:** College of Social Work, University of South Carolina, SC, USA

**Keywords:** OUD, Buprenorphine, OUD Treatment Availability, Buprenorphine Prescriber

## Abstract

**Background:**

Equitable access to medications for opioid use disorder (MOUD) remains a global challenge. Little is known about community geographic, demographic, and socioeconomic characteristics associated with buprenorphine provider availability following removal of the X-waiver. This study examined these associations across U.S. Zip Code Tabulation Areas (ZCTAs).

**Methods:**

We analyzed 32,667 ZCTAs from SAMHSA, the U.S. Census, CDC WONDER, and the Missouri Census Data Center. Outcomes were provider availability (≥1 provider/ZCTA) and provider count. Independent variables included dichotomized measures of urban/rural, racial composition, median household income, educational attainment, unemployment, health insurance coverage, and continuous opioid overdose mortality. Multivariable logistic and negative binomial models were estimated.

**Results:**

30.2% of ZCTAs had at least one provider. Rural ZCTAs had lower provider availability (AOR=0.074, p < .001) and provider counts (IRR=0.070, p < .001). Majority non-White ZCTAs were not significantly associated with either provider availability (AOR=1.1, p = .17) or counts (IRR=1.1, p = .35). Communities with majority less than a bachelor’s degree had lower provider availability (AOR=0.50, p < .001) and counts (IRR=0.53, p < .001). Higher unemployment (AOR=1.5; IRR=1.4, both p < .001), lower health insurance coverage (AOR=1.3; IRR=1.2, p < .001), and higher opioid overdose mortality (AOR=1.3; IRR=1.3, both p < .001) were associated with greater provider availability and counts. Low median household income was not associated with provider availability (AOR=1.0, p = .65) but was associated with higher provider counts (IRR=1.2, p < .001).

**Conclusions:**

Despite the removal of X-waiver, the availability of buprenorphine providers remains limited and uneven. Policies should prioritize equitable distribution of providers to better align treatment capacity with community need, particularly in rural areas.

## Highlights

• About 70% of U.S. ZCTAs lacked a buprenorphine provider.
• Rural ZCTAs had substantially lower provider availability than urban ZCTAs.
• Higher education, unemployment, and lower insurance coverage were associated with provider availability.
• Provider distribution may reflect both healthcare infrastructure and community treatment need.

## 1 Introduction

Opioid use disorder (OUD) and opioid-related harms represent a major public health challenge worldwide, with OUD-related mortality increasing by 12.4% globally between 2017 and 2021 (Zhang et al., 2025). Despite the availability of effective medications for OUD (MOUD), access to treatment remains restricted globally (Nunes et al., 2026). Buprenorphine is a safe and effective medication for OUD, yet it remains underutilized (Andraka-Christou et al., 2023, Shulman et al., 2019). In 2025, over 7.1 million Americans misused opioids, and 4 million met criteria for OUD, yet only 15.7% received evidence-based treatment (SAMHSA, 2026), underscoring the need to expand access to evidence-based OUD treatment.

Historically, the Drug Addiction Treatment Act (DATA) of 2000 hindered workforce growth by requiring buprenorphine providers to obtain an X-waiver including training requirements and patient limits (Silwal et al., 2023). While the 2023 Mainstreaming Addiction Treatment (MAT) Act eliminated the X-waiver, early evidence indicates that gains in treatment initiation and overall treatment engagement have been modest despite increases in buprenorphine providers (Bilden et al., 2025, Chua et al., 2024, Stone et al., 2025). Although previous studies have primarily focused on how expansion of the buprenorphine prescribing workforce has influenced treatment utilization following removal of the X-waiver, less is known about the availability of buprenorphine providers. This study examines the geographic distribution of buprenorphine providers and the community characteristics associated with provider availability following the removal of the X-waiver.

Previous research has identified community characteristics as important correlates of buprenorphine provider availability. Urban-rural location may influence provider availability through differences in healthcare infrastructure (Wang et al., 2025). Community socioeconomic characteristics, including educational attainment, median household income, health insurance coverage, and unemployment—may reflect the economic resources of communities to support the provision of buprenorphine treatment services (Ghertner, 2019, Golan et al., 2023, McBain et al., 2020, Nedjat et al., 2024, Wang et al., 2025). Community racial composition may reflect structural inequities in healthcare access, including provider availability, insurance barriers, discrimination and mistrust (Dunphy et al., 2022, Nedjat et al., 2024). Opioid overdose mortality may identify communities with greater treatment need (McBain et al., 2020). Therefore, this study examined the associations between geographic, socioeconomic, and demographic characteristics and buprenorphine provider availability across U.S. ZIP Code Tabulation Areas (ZCTAs).

## 2 Methods

### 2.1 Study design and sample

We conducted a secondary analysis of publicly available, de-identified cross-sectional data. The unit of analysis was ZCTA, a geographic area approximating U.S. Postal Service ZIP Codes (U.S. Census Bureau, 2023). The sample included 32,667 ZCTAs across the United States and territories. Multivariable analyses used 30,121 ZCTAs with complete data. The Institutional Review Board of the University of South Carolina classified this study as non-human subjects research.

### 2.2 Data sources

Data on buprenorphine provider locations were obtained from the SAMHSA provider database on February 22, 2025, which included providers who consented to public release of their practice information (Nguyen et al., 2022, SAMHSA, 2025). ZCTA-level characteristics came from the 2023 5-year American Community Survey (U.S. Census Bureau, 2025). Geographic classification (urban vs. rural) was based on the 2020 Decennial Census and adapted from U.S. Census rural county definitions: ZCTAs with ≥ 50% rural residents were classified as rural by combining completely rural (100%) and mostly rural (50–99.9%) categories; all others (<50%) were classified as urban (Iyanda et al., 2022). County-level opioid overdose mortality data (2019–2023) were sourced from the CDC WONDER Multiple Cause of Death database (CDC WONDER, 2026), using the CDC’s ICD-10 codes (X40–X44, X60–X64, X85, Y10–Y14; T40.0–T40.4, T40.6). County mortality data were linked to ZCTAs via the Missouri Census Data Center’s MABLE/GeoCorr crosswalk (Missouri Census Data Center, 2018). For ZCTAs spanning multiple counties, population-weighted opioid overdose mortality rates were calculated using the GeoCorr allocation factor, reflecting the proportion of the ZCTA population in each county.

### 2.3 Measures

Two outcome variables were examined. The primary outcome, availability of a buprenorphine provider, was dichotomized (yes/no) to indicate whether a ZCTA had at least one provider. The secondary outcome was the number of providers per ZCTA. Independent variables included location (urban vs. rural), race (majority non-Hispanic White vs. non-White), median household income (below vs. above median), educational attainment (majority bachelor's degree or higher vs. less), unemployment (below vs. above median), and health insurance coverage (below vs. above median). Population-weighted opioid overdose mortality rate was included as a continuous covariate.

### 2.4 Statistical analysis

Descriptive statistics were used to characterize ZCTAs with and without buprenorphine providers. Bivariate associations were tested using Pearson’s chi-square, applying a Bonferroni-adjusted significance threshold of p < .0071 (0.05/7) for seven comparisons. Multivariable logistic regression examined associations between community characteristics and provider availability, reporting adjusted odds ratios (AORs) with 95% confidence intervals (CIs). To complement this, a multivariable negative binomial regression analyzed the number of providers per ZCTA, reporting incidence rate ratios (IRRs) with 95% CIs. Negative binomial regression was chosen due to overdispersion in provider counts. The opioid overdose mortality rate was standardized as a z-score for interpretability. Both models were adjusted for standardized, population-weighted opioid overdose mortality rate and state fixed effects, with robust standard errors clustered by state. Multicollinearity was assessed and was not a concern. All analyses were conducted using Stata/SE version 18.5.

## 3 Results

Among the 32,667 ZCTAs included in the descriptive analysis, 9876 (30.2%) had at least one buprenorphine provider, while 22,791 (69.8%) had none (Table 1).

**Table 1:** Descriptive Characteristics by Buprenorphine Provider Availability.

**Characteristic**	**Total ZCTAs**	**ZCTAs Without Providers**	**ZCTAs With Providers**	***p***
Location				< .001
Rural	20,412 (62.5)	18,451 (90.4)	1961 (9.6)	
Urban	12,255 (37.5)	4340 (35.4)	7915 (64.6)	
Race				< .001
Majority non-white	5450 (16.7)	2744 (50.3)	2706 (49.7)	
Majority non-Hispanic White	27,217 (83.3)	20,047 (73.7)	7170 (26.3)	
Education				< .001
Majority Less than Bachelor’s	28,631 (87.7)	20,970 (73.2)	7661 (26.8)	
Majority Bachelor’s Degree or Higher	4036 (12.3)	1821 (45.1)	2215 (54.9)	
Median Household Income				< .001
Low	14,998 (49.8)	10,874 (72.5)	4124 (27.5)	
High	15,147 (50.2)	9525 (62.9)	5622 (37.1)	
Unemployment Rate				< .001
Low	15,597 (48.5)	12,299 (78.9)	3298 (21.2)	
High	16,532 (51.5)	9976 (60.3)	6556 (39.7)	
Health Insurance Coverage				.007
Low	16,048 (49.7)	11,041 (68.8)	5007 (31.2)	
High	16,261 (50.3)	11,414 (70.2)	4847 (29.8)	
Opioid Overdose Mortality	18.2 (16.8)	16.4 (17.2)	22.4 (15.2)	< .001

Note: Categorical variables are presented as n (%), and opioid overdose mortality is presented as mean (SD). For race and education, the majority category was defined as representing ≥ 50% of the ZCTA population**.** Median household income, unemployment rate, and health insurance coverage were dichotomized at the sample median ($70,005, 4.0%, and 93.8%, respectively). P-values for categorical variables are based on Pearson's chi-square tests comparing ZCTAs with and without buprenorphine providers. Opioid overdose mortality was compared using an independent-samples *t* test. To account for multiple comparisons across seven bivariate analyses, a Bonferroni-adjusted significance threshold of *p* < .0071 (0.05/7) was applied. All bivariate associations remained statistically significant after Bonferroni adjustment.

### 3.1 Urban vs. rural availability

Provider availability was substantially higher in urban ZCTAs than rural ZCTAs (64.6% vs. 9.6%; Table 1). In adjusted analyses, rural ZCTAs had 93% lower odds of having at least one provider (AOR = 0.074, 95% CI [0.065, 0.083], *p* < .001) and 93% lower expected provider counts (IRR = 0.070, 95% CI [0.063, 0.078], *p* < .001) than urban ZCTAs (Table 2).

**Table 2:** Multivariable Models.

	**Logistic**		**Negative Binomial**	
**Characteristics**	**AOR (95% CI)**	***p***	**IRR (95% CI)**	***p***
Location				
Rural	0.074 (0.065–0.083)	< .001	0.07 (0.063–0.078)	< .001
Race				
Majority non-White	1.1 (0.95–1.4)	.17	1.1 (0.92–1.2)	.35
Education				
Majority Less than Bachelor’s	0.50 (0.43–0.58)	< .001	0.53 (0.47–0.59)	< .001
Median Household Income				
Low	1.0 (0.91–1.2)	.65	1.2 (1.1–1.3)	< .001
Unemployment Rate				
High	1.5 (1.4–1.7)	< .001	1.4 (1.3–1.6)	< .001
Health Insurance Coverage				
Low	1.3 (1.1–1.4)	< .001	1.2 (1.1–1.4)	< .001
Opioid Overdose Mortality	1.3 (1.2–1.4)	< .001	1.3 (1.2–1.4)	< .001
State Fixed Effects	Included		Included	

Note: AOR =  adjusted odds ratio; IRR =  incidence rate ratio. Reference categories were urban (location), majority non-Hispanic White (race), majority bachelor's degree or higher (education), high median household income, low unemployment rate, and high health insurance coverage. Both models were adjusted for standardized, population-weighted opioid overdose mortality rate and state fixed effects; state-specific coefficients are not shown. State fixed effects were replaced with Medicaid expansion status, which yielded an OR of 1.1 (95% CI [0.84, 1.5], *p* = .46) and an IRR of 1.4 (95% CI [1.1, 1.7], *p* = .003). State fixed effects were retained to account for unobserved state-level differences.

### 3.2 Availability by race

Nearly half of majority non-White ZCTAs had at least one provider compared with one-quarter of majority non-Hispanic White ZCTAs (49.7% vs. 26.3%; Table 1). However, majority non-White ZCTAs did not differ significantly from majority non-Hispanic White ZCTAs in either the odds of having at least one provider (AOR = 1.1, 95% CI [0.95, 1.4], *p* = .17; Table 2) or expected provider counts (IRR = 1.1, 95% CI [0.92, 1.2], *p* = .35; Table 2).

### 3.3 Availability by socioeconomic characteristics

Descriptively, 37.1% of high-income ZCTAs had at least one provider compared with 27.5% of low-income ZCTAs (Table 1). Although low median household income was not significantly associated with the odds of provider availability (AOR = 1.0, 95% CI [0.91, 1.2], *p* = .65; Table 2), low-income ZCTAs had 20% higher expected provider counts than high-income ZCTAs (IRR = 1.2, 95% CI [1.1, 1.3], *p* < .001; Table 2).

Educational attainment was associated with both outcomes. ZCTAs in which the majority of residents had less than a bachelor's degree had 50% lower odds of having at least one provider (AOR = 0.50, 95% CI [0.43, 0.58], *p* < .001) and 47% lower expected provider counts (IRR = 0.53, 95% CI [0.47, 0.59], *p* < .001) than ZCTAs in which the majority had a bachelor's degree or higher.

High-unemployment ZCTAs had 50% higher odds of having at least one provider (AOR = 1.5, 95% CI [1.4, 1.7], *p* < .001; Table 2) and 40% higher expected provider counts (IRR = 1.4, 95% CI [1.3, 1.6], *p* < .001; Table 2) than low-unemployment ZCTAs. Likewise, ZCTAs with low health insurance coverage had 30% higher odds of having at least one provider (AOR = 1.3, 95% CI [1.1, 1.4], *p* < .001; Table 2) and 20% higher expected provider counts (IRR = 1.2, 95% CI [1.1, 1.4], *p* < .001; Table 2) than ZCTAs with high health insurance coverage.

#### 3.3.1 Availability by opioid overdose mortality

A one-standard-deviation increase in opioid overdose mortality was associated with 30% higher odds of having at least one buprenorphine provider (AOR = 1.3, 95% CI [1.2, 1.4], *p* < .001; Table 2) and 30% higher expected provider counts (IRR = 1.3, 95% CI [1.2, 1.4], *p* < .001; Table 2).

## 4 Discussion

Our study has several key findings. First, over two-thirds of ZCTAs lacked providers despite an increase from 38,684 providers in January 2022–53,635 in December 2023 (Chua et al., 2024). Second, urban ZCTAs had significantly higher provider availability than rural ones. Although only 37.5% of ZCTAs were urban and 62.5% rural, urban ZCTAs with providers outnumbered rural ZCTAs with providers by about four to one. This aligns with prior research showing persistent shortages of buprenorphine providers in rural areas (McBain et al., 2020).

Our third finding concerned racial composition. Nearly half of majority non-White ZCTAs had at least one provider, compared to about one-quarter of majority non-Hispanic White ZCTAs. While this aligns with a national study showing greater provider presence in racially diverse ZIP codes (Hirchak et al., 2022), our multivariable analyses found no independent association between racial composition and provider availability. This contrasts with studies reporting lower buprenorphine treatment use among racial and ethnic minorities (Dunphy et al., 2022, Nedjat et al., 2024), underscoring the difference between provider availability and realized access. The observed descriptive difference may reflect geographic context, as majority non-White ZCTAs were more urban than majority non-Hispanic White ZCTAs (67% vs. 33%), consistent with evidence that the association between racial/ethnic composition and provider availability may vary by urban-rural status (Hirchak et al., 2022). Future research should distinguish provider availability from treatment utilization and examine how race/ethnicity and urban-rural location jointly shape access to buprenorphine treatment.

Our findings on community socioeconomic characteristics showed mixed results compared to prior research. Median household income was not independently linked to the presence of at least one provider, but higher provider counts in lower-income communities contrast with studies showing greater buprenorphine access in higher-income areas (Golan et al., 2023, Nedjat et al., 2024). Similarly, greater provider availability in communities with higher unemployment contrasts with Ghertner (2019), who reported lower treatment capacity in such counties. Health insurance coverage findings were also mixed: greater provider availability in areas with lower insurance coverage aligns with Ghertner (2019) but contradicts Wang et al. (2025), who found fewer providers where uninsured rates were higher. These discrepancies likely reflect different community contexts. Higher educational attainment may signal stronger healthcare infrastructure and workforce capacity, while higher unemployment, lower insurance coverage, and greater overdose rates indicate greater treatment need. These factors often coexist in urban areas, where healthcare systems, educational institutions, and vulnerable populations concentrate. Prior research supports this, showing provider concentration in urban areas and specialties more prevalent in counties with higher education levels (Naylor et al., 2019, Odisho et al., 2009, Tran et al., 2024). McBain et al. (2020) similarly found that growth in waivered buprenorphine prescribers was concentrated in metropolitan counties with higher education, physician supply, and opioid overdose deaths.

These findings suggest that buprenorphine provider distribution may reflect both healthcare infrastructure and community treatment needs. However, provider availability should not be interpreted as evidence that treatment needs are being adequately met. The absence of providers in about 70% of ZCTAs and significant urban-rural disparities indicate that federal deregulation alone may be insufficient to ensure equitable access to evidence-based OUD treatment. These findings also have broader international relevance, as access to MOUD remains inadequate globally and shortages of treatment providers continue to constrain treatment availability, particularly in rural and underserved communities. Future research should examine whether buprenorphine provider presence in economically vulnerable communities corresponds to the level of treatment need and whether these communities are receiving adequate and equitable access to OUD treatment services.

This study has several limitations. The SAMHSA buprenorphine provider database does not capture all buprenorphine providers but only those who chose to list their practice information. Listed providers may also not have been actively prescribing or accepting new patients, potentially overestimating treatment availability within a ZCTA. Telehealth may further allow individuals to receive buprenorphine from outside their ZCTA, so geographic provider presence may not fully reflect treatment availability in a community. Unmeasured factors, such as provider practice characteristics and healthcare infrastructure, may also have influenced the observed associations.

## CRediT authorship contribution statement

**Seay Kristen:** Writing – review & editing, Validation, Supervision, Methodology. **Sadia Jehan:** Writing – original draft, Software, Methodology, Formal analysis, Data curation, Conceptualization.

## Funding

This research did not receive any specific grant from funding agencies in the public, commercial, or non-profit sectors.

## Declaration of Competing Interest

The authors declare that they have no known competing financial interests or personal relationships that could have appeared to influence the work reported in this paper.

## Data Availability

The data that support the findings of this study are available from the corresponding author upon request.
